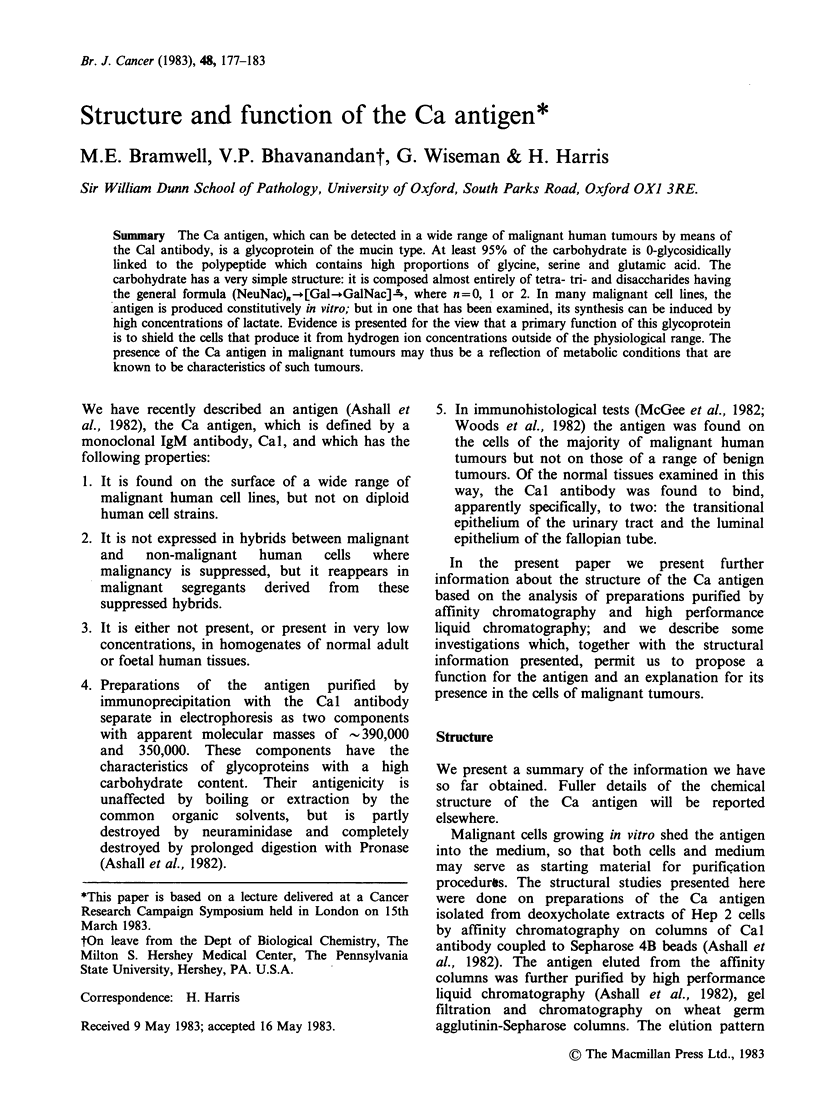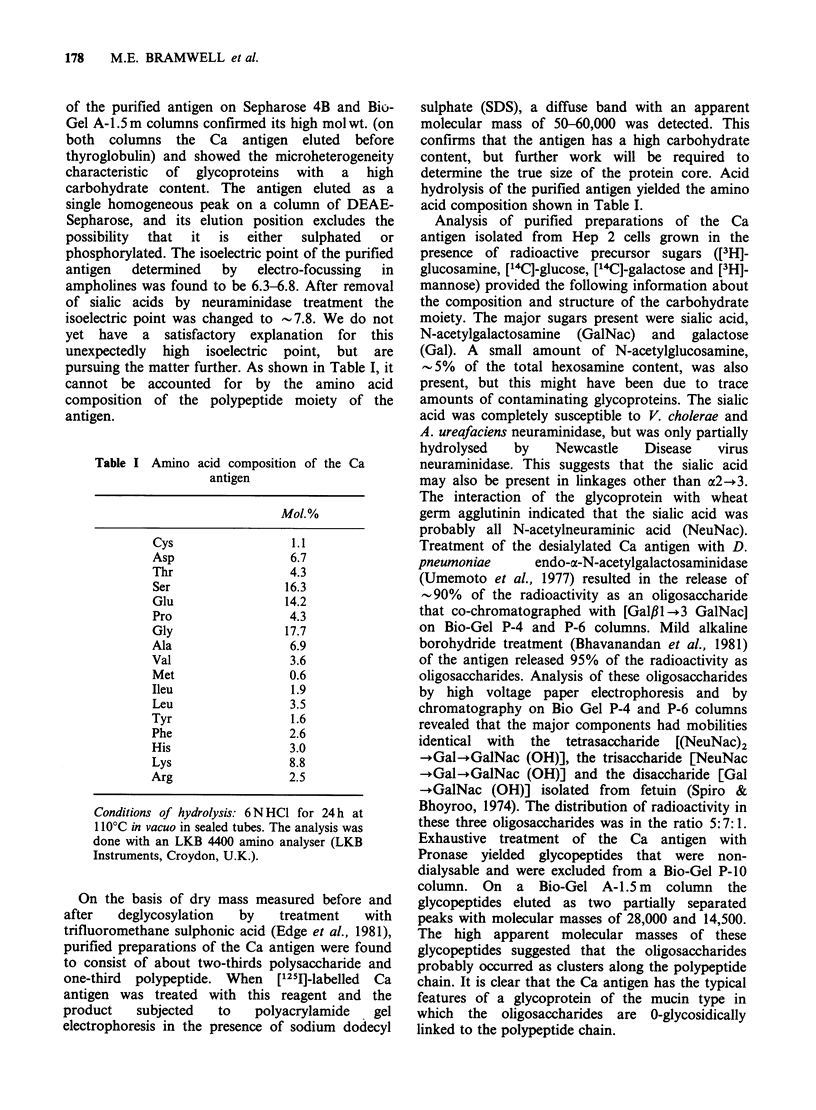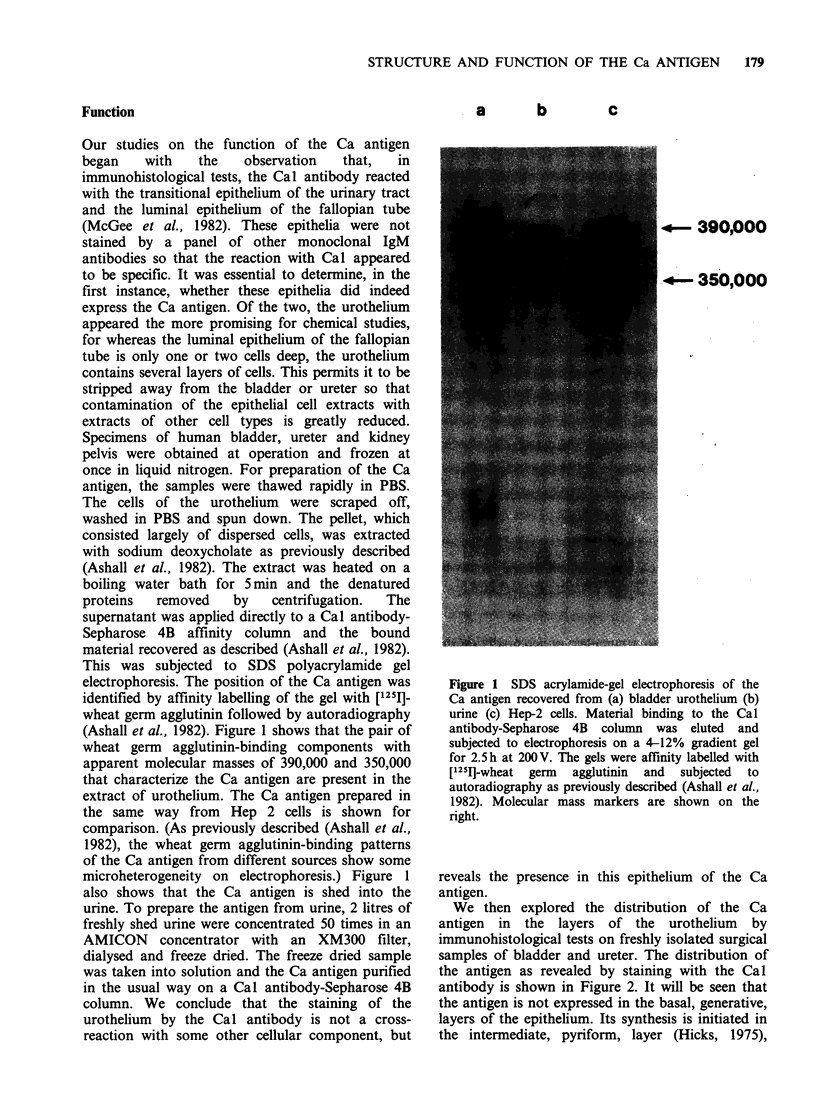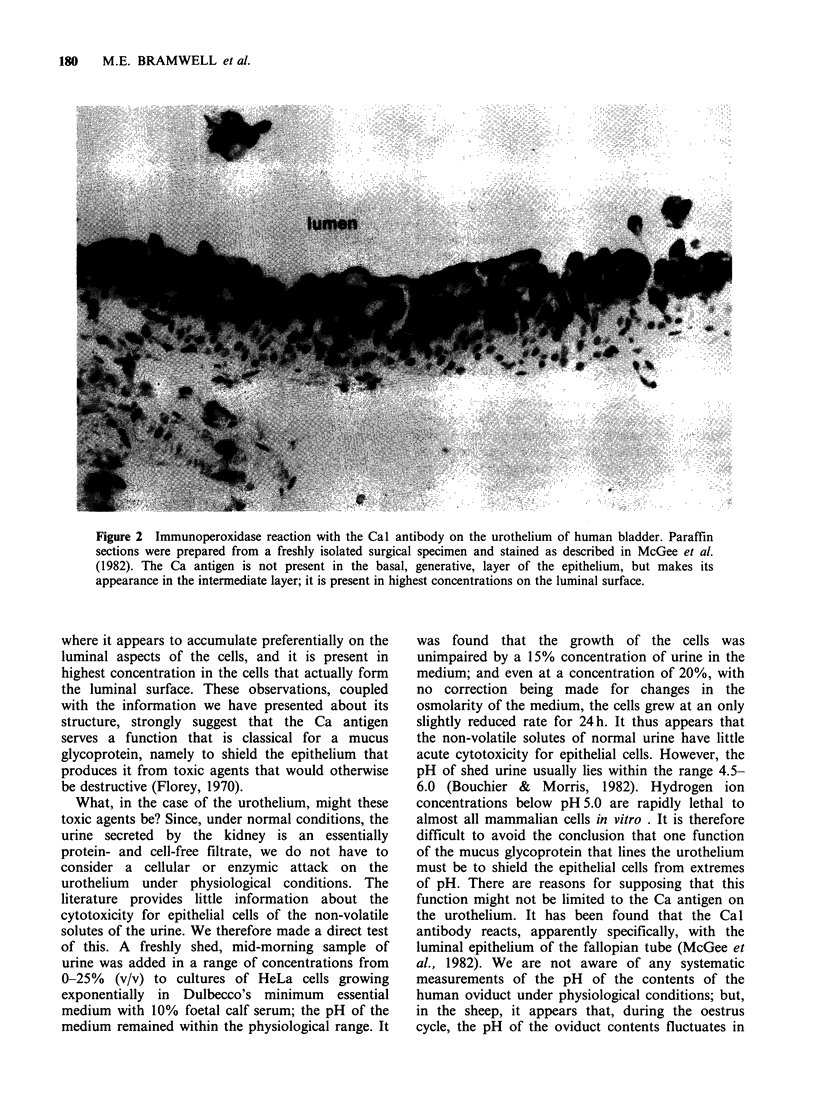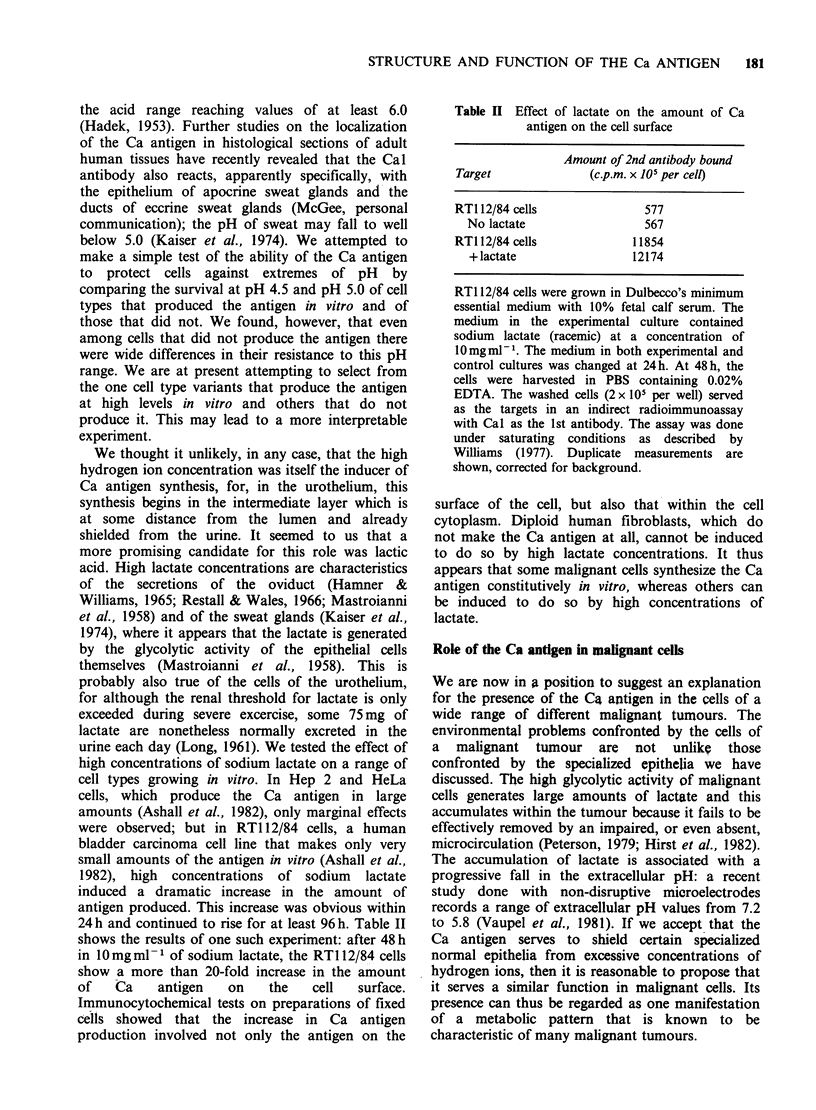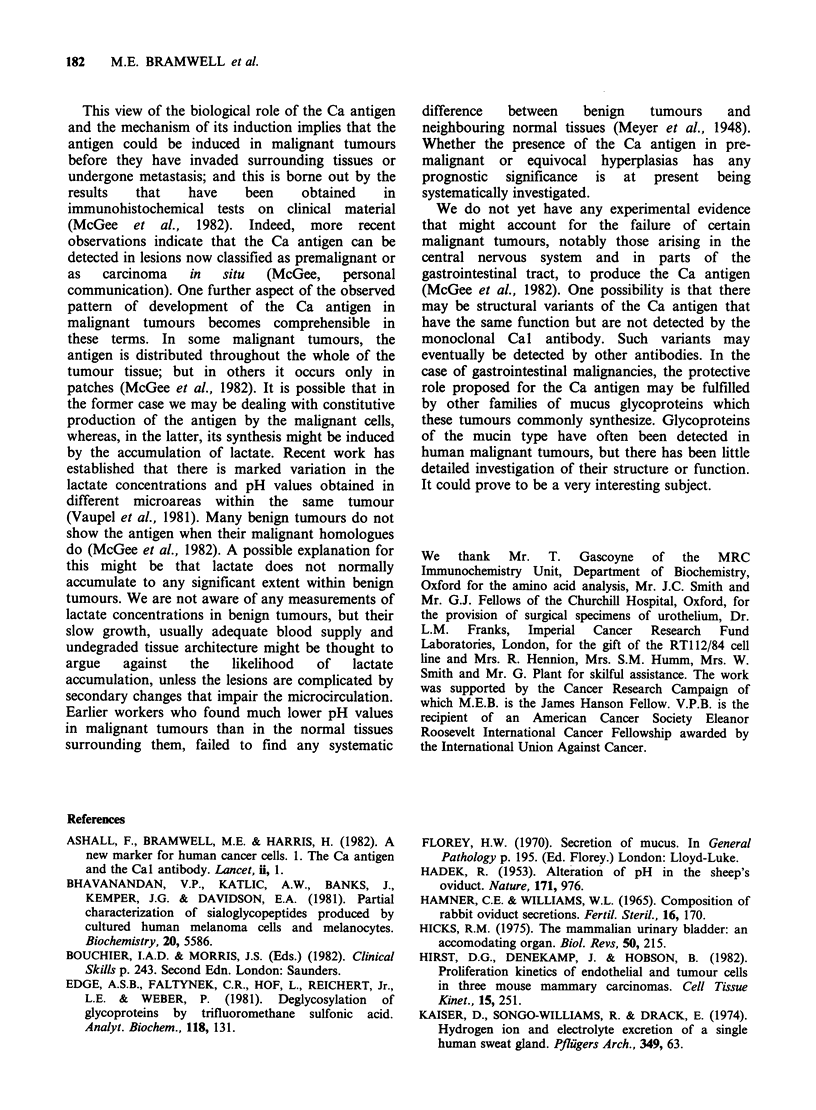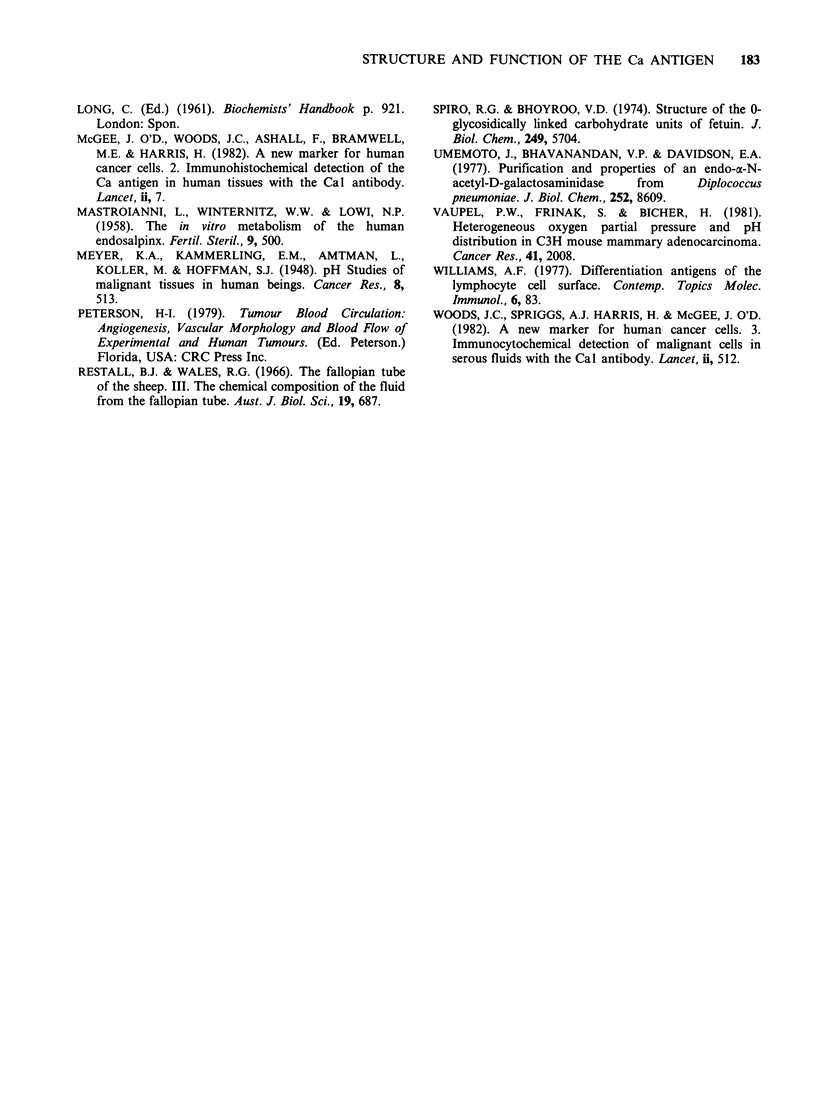# Structure and function of the Ca antigen.

**DOI:** 10.1038/bjc.1983.173

**Published:** 1983-08

**Authors:** M. E. Bramwell, V. P. Bhavanandan, G. Wiseman, H. Harris

## Abstract

**Images:**


*Br. J. Cancer* (1983), **48**, 177–183

### Structure and function of the Ca antigen*

M.E. Bramwell, V.P. Bhavanandan†, G. Wiseman & H. Harris

*Sir William Dunn School of Pathology, University of Oxford, South Parks Road, Oxford OX1 3RE.*

**Summary** The Ca antigen, which can be detected in a wide range of malignant human tumours by means of the Cal antibody, is a glycoprotein of the mucin type. At least 95% of the carbohydrate is 0-glycosidically linked to the polypeptide which contains high proportions of glycine, serine and glutamic acid. The carbohydrate has a very simple structure: it is composed almost entirely of tetra- tri- and disaccharides having the general formula $(\text{NeuNac})_n \rightarrow [\text{Gal} \rightarrow \text{GalNac}] \xrightarrow{\alpha}$, where $n = 0$, 1 or 2. In many malignant cell lines, the antigen is produced constitutively *in vitro;* but in one that has been examined, its synthesis can be induced by high concentrations of lactate. Evidence is presented for the view that a primary function of this glycoprotein is to shield the cells that produce it from hydrogen ion concentrations outside of the physiological range. The presence of the Ca antigen in malignant tumours may thus be a reflection of metabolic conditions that are known to be characteristics of such tumours.

We have recently described an antigen (Ashall *et al.,* 1982), the Ca antigen, which is defined by a monoclonal IgM antibody, Ca1, and which has the following properties:

1. It is found on the surface of a wide range of malignant human cell lines, but not on diploid human cell strains.
2. It is not expressed in hybrids between malignant and non-malignant human cells where malignancy is suppressed, but it reappears in malignant segregants derived from these suppressed hybrids.
3. It is either not present, or present in very low concentrations, in homogenates of normal adult or foetal human tissues.
4. Preparations of the antigen purified by immunoprecipitation with the Cal antibody separate in electrophoresis as two components with apparent molecular masses of ~390,000 and 350,000. These components have the characteristics of glycoproteins with a high carbohydrate content. Their antigenicity is unaffected by boiling or extraction by the common organic solvents, but is partly destroyed by neuraminidase and completely destroyed by prolonged digestion with Pronase (Ashall *et al.,* 1982).
5. In immunohistological tests (McGee *et al.,* 1982; Woods *et al.,* 1982) the antigen was found on the cells of the majority of malignant human tumours but not on those of a range of benign tumours. Of the normal tissues examined in this way, the Cal antibody was found to bind, apparently specifically, to two: the transitional epithelium of the urinary tract and the luminal epithelium of the fallopian tube.

In the present paper we present further information about the structure of the Ca antigen based on the analysis of preparations purified by affinity chromatography and high performance liquid chromatography; and we describe some investigations which, together with the structural information presented, permit us to propose a function for the antigen and an explanation for its presence in the cells of malignant tumours.

### Structure

We present a summary of the information we have so far obtained. Fuller details of the chemical structure of the Ca antigen will be reported elsewhere.

Malignant cells growing *in vitro* shed the antigen into the medium, so that both cells and medium may serve as starting material for purification procedures. The structural studies presented here were done on preparations of the Ca antigen isolated from deoxycholate extracts of Hep 2 cells by affinity chromatography on columns of Cal antibody coupled to Sepharose 4B beads (Ashall *et al.,* 1982). The antigen eluted from the affinity columns was further purified by high performance liquid chromatography (Ashall *et al.,* 1982), gel filtration and chromatography on wheat germ agglutinin-Sepharose columns. The elution pattern

*This paper is based on a lecture delivered at a Cancer Research Campaign Symposium held in London on 15th March 1983.

†On leave from the Dept of Biological Chemistry, The Milton S. Hershey Medical Center, The Pennsylvania State University, Hershey, PA. U.S.A.

Correspondence: H. Harris

Received 9 May 1983; accepted 16 May 1983.

© The Macmillan Press Ltd., 1983

of the purified antigen on Sepharose 4B and Bio-Gel A-1.5 m columns confirmed its high mol wt. (on both columns the Ca antigen eluted before thyroglobulin) and showed the microheterogeneity characteristic of glycoproteins with a high carbohydrate content. The antigen eluted as a single homogeneous peak on a column of DEAE-Sepharose, and its elution position excludes the possibility that it is either sulphated or phosphorylated. The isoelectric point of the purified antigen determined by electro-focussing in ampholines was found to be 6.3–6.8. After removal of sialic acids by neuraminidase treatment the isoelectric point was changed to ~7.8. We do not yet have a satisfactory explanation for this unexpectedly high isoelectric point, but are pursuing the matter further. As shown in Table I, it cannot be accounted for by the amino acid composition of the polypeptide moiety of the antigen.

**Table I** Amino acid composition of the Ca antigen

| | *Mol.%* |
|---|---|
| Cys | 1.1 |
| Asp | 6.7 |
| Thr | 4.3 |
| Ser | 16.3 |
| Glu | 14.2 |
| Pro | 4.3 |
| Gly | 17.7 |
| Ala | 6.9 |
| Val | 3.6 |
| Met | 0.6 |
| Ileu | 1.9 |
| Leu | 3.5 |
| Tyr | 1.6 |
| Phe | 2.6 |
| His | 3.0 |
| Lys | 8.8 |
| Arg | 2.5 |

*Conditions of hydrolysis:* 6 N HCl for 24 h at 110°C *in vacuo* in sealed tubes. The analysis was done with an LKB 4400 amino analyser (LKB Instruments, Croydon, U.K.).

On the basis of dry mass measured before and after deglycosylation by treatment with trifluoromethane sulphonic acid (Edge *et al.*, 1981), purified preparations of the Ca antigen were found to consist of about two-thirds polysaccharide and one-third polypeptide. When [$^{125}$I]-labelled Ca antigen was treated with this reagent and the product subjected to polyacrylamide gel electrophoresis in the presence of sodium dodecyl sulphate (SDS), a diffuse band with an apparent molecular mass of 50–60,000 was detected. This confirms that the antigen has a high carbohydrate content, but further work will be required to determine the true size of the protein core. Acid hydrolysis of the purified antigen yielded the amino acid composition shown in Table I.

Analysis of purified preparations of the Ca antigen isolated from Hep 2 cells grown in the presence of radioactive precursor sugars ([$^{3}$H]-glucosamine, [$^{14}$C]-glucose, [$^{14}$C]-galactose and [$^{3}$H]-mannose) provided the following information about the composition and structure of the carbohydrate moiety. The major sugars present were sialic acid, N-acetylgalactosamine (GalNac) and galactose (Gal). A small amount of N-acetylglucosamine, ~5% of the total hexosamine content, was also present, but this might have been due to trace amounts of contaminating glycoproteins. The sialic acid was completely susceptible to *V. cholerae* and *A. ureafaciens* neuraminidase, but was only partially hydrolysed by Newcastle Disease virus neuraminidase. This suggests that the sialic acid may also be present in linkages other than $\alpha 2 \rightarrow 3$. The interaction of the glycoprotein with wheat germ agglutinin indicated that the sialic acid was probably all N-acetylneuraminic acid (NeuNac). Treatment of the desialylated Ca antigen with *D. pneumoniae* endo-α-N-acetylgalactosaminidase (Umemoto *et al.*, 1977) resulted in the release of ~90% of the radioactivity as an oligosaccharide that co-chromatographed with [Gal$\beta$1→3 GalNac] on Bio-Gel P-4 and P-6 columns. Mild alkaline borohydride treatment (Bhavanandan *et al.*, 1981) of the antigen released 95% of the radioactivity as oligosaccharides. Analysis of these oligosaccharides by high voltage paper electrophoresis and by chromatography on Bio Gel P-4 and P-6 columns revealed that the major components had mobilities identical with the tetrasaccharide [$(NeuNac)_2$ →Gal→GalNac (OH)], the trisaccharide [NeuNac →Gal→GalNac (OH)] and the disaccharide [Gal →GalNac (OH)] isolated from fetuin (Spiro & Bhoyroo, 1974). The distribution of radioactivity in these three oligosaccharides was in the ratio 5:7:1. Exhaustive treatment of the Ca antigen with Pronase yielded glycopeptides that were non-dialysable and were excluded from a Bio-Gel P-10 column. On a Bio-Gel A-1.5 m column the glycopeptides eluted as two partially separated peaks with molecular masses of 28,000 and 14,500. The high apparent molecular masses of these glycopeptides suggested that the oligosaccharides probably occurred as clusters along the polypeptide chain. It is clear that the Ca antigen has the typical features of a glycoprotein of the mucin type in which the oligosaccharides are 0-glycosidically linked to the polypeptide chain.

### Function

Our studies on the function of the Ca antigen began with the observation that, in immunohistological tests, the Ca1 antibody reacted with the transitional epithelium of the urinary tract and the luminal epithelium of the fallopian tube (McGee *et al.,* 1982). These epithelia were not stained by a panel of other monoclonal IgM antibodies so that the reaction with Ca1 appeared to be specific. It was essential to determine, in the first instance, whether these epithelia did indeed express the Ca antigen. Of the two, the urothelium appeared the more promising for chemical studies, for whereas the luminal epithelium of the fallopian tube is only one or two cells deep, the urothelium contains several layers of cells. This permits it to be stripped away from the bladder or ureter so that contamination of the epithelial cell extracts with extracts of other cell types is greatly reduced. Specimens of human bladder, ureter and kidney pelvis were obtained at operation and frozen at once in liquid nitrogen. For preparation of the Ca antigen, the samples were thawed rapidly in PBS. The cells of the urothelium were scraped off, washed in PBS and spun down. The pellet, which consisted largely of dispersed cells, was extracted with sodium deoxycholate as previously described (Ashall *et al.,* 1982). The extract was heated on a boiling water bath for 5 min and the denatured proteins removed by centrifugation. The supernatant was applied directly to a Ca1 antibody-Sepharose 4B affinity column and the bound material recovered as described (Ashall *et al.,* 1982). This was subjected to SDS polyacrylamide gel electrophoresis. The position of the Ca antigen was identified by affinity labelling of the gel with [$^{125}$I]-wheat germ agglutinin followed by autoradiography (Ashall *et al.,* 1982). Figure 1 shows that the pair of wheat germ agglutinin-binding components with apparent molecular masses of 390,000 and 350,000 that characterize the Ca antigen are present in the extract of urothelium. The Ca antigen prepared in the same way from Hep 2 cells is shown for comparison. (As previously described (Ashall *et al.,* 1982), the wheat germ agglutinin-binding patterns of the Ca antigen from different sources show some microheterogeneity on electrophoresis.) Figure 1 also shows that the Ca antigen is shed into the urine. To prepare the antigen from urine, 2 litres of freshly shed urine were concentrated 50 times in an AMICON concentrator with an XM300 filter, dialysed and freeze dried. The freeze dried sample was taken into solution and the Ca antigen purified in the usual way on a Ca1 antibody-Sepharose 4B column. We conclude that the staining of the urothelium by the Ca1 antibody is not a cross-reaction with some other cellular component, but reveals the presence in this epithelium of the Ca antigen.

**Figure 1** SDS acrylamide-gel electrophoresis of the Ca antigen recovered from (a) bladder urothelium (b) urine (c) Hep-2 cells. Material binding to the Ca1 antibody-Sepharose 4B column was eluted and subjected to electrophoresis on a 4–12% gradient gel for 2.5 h at 200 V. The gels were affinity labelled with [$^{125}$I]-wheat germ agglutinin and subjected to autoradiography as previously described (Ashall *et al.,* 1982). Molecular mass markers are shown on the right.

We then explored the distribution of the Ca antigen in the layers of the urothelium by immunohistological tests on freshly isolated surgical samples of bladder and ureter. The distribution of the antigen as revealed by staining with the Ca1 antibody is shown in Figure 2. It will be seen that the antigen is not expressed in the basal, generative, layers of the epithelium. Its synthesis is initiated in the intermediate, pyriform, layer (Hicks, 1975),

**Figure 2** Immunoperoxidase reaction with the Ca1 antibody on the urothelium of human bladder. Paraffin sections were prepared from a freshly isolated surgical specimen and stained as described in McGee *et al.* (1982). The Ca antigen is not present in the basal, generative, layer of the epithelium, but makes its appearance in the intermediate layer; it is present in highest concentrations on the luminal surface.

where it appears to accumulate preferentially on the luminal aspects of the cells, and it is present in highest concentration in the cells that actually form the luminal surface. These observations, coupled with the information we have presented about its structure, strongly suggest that the Ca antigen serves a function that is classical for a mucus glycoprotein, namely to shield the epithelium that produces it from toxic agents that would otherwise be destructive (Florey, 1970).

What, in the case of the urothelium, might these toxic agents be? Since, under normal conditions, the urine secreted by the kidney is an essentially protein- and cell-free filtrate, we do not have to consider a cellular or enzymic attack on the urothelium under physiological conditions. The literature provides little information about the cytotoxicity for epithelial cells of the non-volatile solutes of the urine. We therefore made a direct test of this. A freshly shed, mid-morning sample of urine was added in a range of concentrations from 0–25% (v/v) to cultures of HeLa cells growing exponentially in Dulbecco's minimum essential medium with 10% foetal calf serum; the pH of the medium remained within the physiological range. It was found that the growth of the cells was unimpaired by a 15% concentration of urine in the medium; and even at a concentration of 20%, with no correction being made for changes in the osmolarity of the medium, the cells grew at an only slightly reduced rate for 24 h. It thus appears that the non-volatile solutes of normal urine have little acute cytotoxicity for epithelial cells. However, the pH of shed urine usually lies within the range 4.5–6.0 (Bouchier & Morris, 1982). Hydrogen ion concentrations below pH 5.0 are rapidly lethal to almost all mammalian cells *in vitro* . It is therefore difficult to avoid the conclusion that one function of the mucus glycoprotein that lines the urothelium must be to shield the epithelial cells from extremes of pH. There are reasons for supposing that this function might not be limited to the Ca antigen on the urothelium. It has been found that the Ca1 antibody reacts, apparently specifically, with the luminal epithelium of the fallopian tube (McGee *et al.*, 1982). We are not aware of any systematic measurements of the pH of the contents of the human oviduct under physiological conditions; but, in the sheep, it appears that, during the oestrus cycle, the pH of the oviduct contents fluctuates in the acid range reaching values of at least 6.0 (Hadek, 1953). Further studies on the localization of the Ca antigen in histological sections of adult human tissues have recently revealed that the Ca1 antibody also reacts, apparently specifically, with the epithelium of apocrine sweat glands and the ducts of eccrine sweat glands (McGee, personal communication); the pH of sweat may fall to well below 5.0 (Kaiser *et al.*, 1974). We attempted to make a simple test of the ability of the Ca antigen to protect cells against extremes of pH by comparing the survival at pH 4.5 and pH 5.0 of cell types that produced the antigen *in vitro* and of those that did not. We found, however, that even among cells that did not produce the antigen there were wide differences in their resistance to this pH range. We are at present attempting to select from the one cell type variants that produce the antigen at high levels *in vitro* and others that do not produce it. This may lead to a more interpretable experiment.

We thought it unlikely, in any case, that the high hydrogen ion concentration was itself the inducer of Ca antigen synthesis, for, in the urothelium, this synthesis begins in the intermediate layer which is at some distance from the lumen and already shielded from the urine. It seemed to us that a more promising candidate for this role was lactic acid. High lactate concentrations are characteristics of the secretions of the oviduct (Hamner & Williams, 1965; Restall & Wales, 1966; Mastroianni *et al.*, 1958) and of the sweat glands (Kaiser *et al.*, 1974), where it appears that the lactate is generated by the glycolytic activity of the epithelial cells themselves (Mastroianni *et al.*, 1958). This is probably also true of the cells of the urothelium, for although the renal threshold for lactate is only exceeded during severe excercise, some 75 mg of lactate are nonetheless normally excreted in the urine each day (Long, 1961). We tested the effect of high concentrations of sodium lactate on a range of cell types growing *in vitro*. In Hep 2 and HeLa cells, which produce the Ca antigen in large amounts (Ashall *et al.*, 1982), only marginal effects were observed; but in RT112/84 cells, a human bladder carcinoma cell line that makes only very small amounts of the antigen *in vitro* (Ashall *et al.*, 1982), high concentrations of sodium lactate induced a dramatic increase in the amount of antigen produced. This increase was obvious within 24 h and continued to rise for at least 96 h. Table II shows the results of one such experiment: after 48 h in 10 mg $ml^{-1}$ of sodium lactate, the RT112/84 cells show a more than 20-fold increase in the amount of Ca antigen on the cell surface. Immunocytochemical tests on preparations of fixed cells showed that the increase in Ca antigen production involved not only the antigen on the surface of the cell, but also that within the cell cytoplasm. Diploid human fibroblasts, which do not make the Ca antigen at all, cannot be induced to do so by high lactate concentrations. It thus appears that some malignant cells synthesize the Ca antigen constitutively *in vitro,* whereas others can be induced to do so by high concentrations of lactate.

**Table II** Effect of lactate on the amount of Ca antigen on the cell surface

| *Target* | *Amount of 2nd antibody bound (c.p.m. × $10^5$ per cell)* |
|---|---|
| RT112/84 cells | 577 |
| No lactate | 567 |
| RT112/84 cells | 11854 |
| +lactate | 12174 |

RT112/84 cells were grown in Dulbecco's minimum essential medium with 10% fetal calf serum. The medium in the experimental culture contained sodium lactate (racemic) at a concentration of 10 mg $ml^{-1}$. The medium in both experimental and control cultures was changed at 24 h. At 48 h, the cells were harvested in PBS containing 0.02% EDTA. The washed cells ($2 \times 10^5$ per well) served as the targets in an indirect radioimmunoassay with Ca1 as the 1st antibody. The assay was done under saturating conditions as described by Williams (1977). Duplicate measurements are shown, corrected for background.

### Role of the Ca antigen in malignant cells

We are now in a position to suggest an explanation for the presence of the Ca antigen in the cells of a wide range of different malignant tumours. The environmental problems confronted by the cells of a malignant tumour are not unlike those confronted by the specialized epithelia we have discussed. The high glycolytic activity of malignant cells generates large amounts of lactate and this accumulates within the tumour because it fails to be effectively removed by an impaired, or even absent, microcirculation (Peterson, 1979; Hirst *et al.*, 1982). The accumulation of lactate is associated with a progressive fall in the extracellular pH: a recent study done with non-disruptive microelectrodes records a range of extracellular pH values from 7.2 to 5.8 (Vaupel *et al.*, 1981). If we accept that the Ca antigen serves to shield certain specialized normal epithelia from excessive concentrations of hydrogen ions, then it is reasonable to propose that it serves a similar function in malignant cells. Its presence can thus be regarded as one manifestation of a metabolic pattern that is known to be characteristic of many malignant tumours.

This view of the biological role of the Ca antigen and the mechanism of its induction implies that the antigen could be induced in malignant tumours before they have invaded surrounding tissues or undergone metastasis; and this is borne out by the results that have been obtained in immunohistochemical tests on clinical material (McGee *et al.*, 1982). Indeed, more recent observations indicate that the Ca antigen can be detected in lesions now classified as premalignant or as carcinoma *in situ* (McGee, personal communication). One further aspect of the observed pattern of development of the Ca antigen in malignant tumours becomes comprehensible in these terms. In some malignant tumours, the antigen is distributed throughout the whole of the tumour tissue; but in others it occurs only in patches (McGee *et al.*, 1982). It is possible that in the former case we may be dealing with constitutive production of the antigen by the malignant cells, whereas, in the latter, its synthesis might be induced by the accumulation of lactate. Recent work has established that there is marked variation in the lactate concentrations and pH values obtained in different microareas within the same tumour (Vaupel *et al.*, 1981). Many benign tumours do not show the antigen when their malignant homologues do (McGee *et al.*, 1982). A possible explanation for this might be that lactate does not normally accumulate to any significant extent within benign tumours. We are not aware of any measurements of lactate concentrations in benign tumours, but their slow growth, usually adequate blood supply and undegraded tissue architecture might be thought to argue against the likelihood of lactate accumulation, unless the lesions are complicated by secondary changes that impair the microcirculation. Earlier workers who found much lower pH values in malignant tumours than in the normal tissues surrounding them, failed to find any systematic difference between benign tumours and neighbouring normal tissues (Meyer *et al.*, 1948). Whether the presence of the Ca antigen in premalignant or equivocal hyperplasias has any prognostic significance is at present being systematically investigated.

We do not yet have any experimental evidence that might account for the failure of certain malignant tumours, notably those arising in the central nervous system and in parts of the gastrointestinal tract, to produce the Ca antigen (McGee *et al.*, 1982). One possibility is that there may be structural variants of the Ca antigen that have the same function but are not detected by the monoclonal Ca1 antibody. Such variants may eventually be detected by other antibodies. In the case of gastrointestinal malignancies, the protective role proposed for the Ca antigen may be fulfilled by other families of mucus glycoproteins which these tumours commonly synthesize. Glycoproteins of the mucin type have often been detected in human malignant tumours, but there has been little detailed investigation of their structure or function. It could prove to be a very interesting subject.

We thank Mr. T. Gascoyne of the MRC Immunochemistry Unit, Department of Biochemistry, Oxford for the amino acid analysis, Mr. J.C. Smith and Mr. G.J. Fellows of the Churchill Hospital, Oxford, for the provision of surgical specimens of urothelium, Dr. L.M. Franks, Imperial Cancer Research Fund Laboratories, London, for the gift of the RT112/84 cell line and Mrs. R. Hennion, Mrs. S.M. Humm, Mrs. W. Smith and Mr. G. Plant for skilful assistance. The work was supported by the Cancer Research Campaign of which M.E.B. is the James Hanson Fellow. V.P.B. is the recipient of an American Cancer Society Eleanor Roosevelt International Cancer Fellowship awarded by the International Union Against Cancer.

### References

ASHALL, F., BRAMWELL, M.E. & HARRIS, H. (1982). A new marker for human cancer cells. 1. The Ca antigen and the Ca1 antibody. *Lancet*, **ii**, 1.

BHAVANANDAN, V.P., KATLIC, A.W., BANKS, J., KEMPER, J.G. & DAVIDSON, E.A. (1981). Partial characterization of sialoglycopeptides produced by cultured human melanoma cells and melanocytes. *Biochemistry*, **20**, 5586.

BOUCHIER, I.A.D. & MORRIS, J.S. (Eds.) (1982). *Clinical Skills* p. 243. Second Edn. London: Saunders.

EDGE, A.S.B., FALTYNEK, C.R., HOF, L., REICHERT, Jr., L.E. & WEBER, P. (1981). Deglycosylation of glycoproteins by trifluoromethane sulfonic acid. *Analyt. Biochem.*, **118**, 131.

FLOREY, H.W. (1970). Secretion of mucus. In *General Pathology* p. 195. (Ed. Florey.) London: Lloyd-Luke.

HADEK, R. (1953). Alteration of pH in the sheep's oviduct. *Nature*, **171**, 976.

HAMNER, C.E. & WILLIAMS, W.L. (1965). Composition of rabbit oviduct secretions. *Fertil. Steril.*, **16**, 170.

HICKS, R.M. (1975). The mammalian urinary bladder: an accomodating organ. *Biol. Revs*, **50**, 215.

HIRST, D.G., DENEKAMP, J. & HOBSON, B. (1982). Proliferation kinetics of endothelial and tumour cells in three mouse mammary carcinomas. *Cell Tissue Kinet.*, **15**, 251.

KAISER, D., SONGO-WILLIAMS, R. & DRACK, E. (1974). Hydrogen ion and electrolyte excretion of a single human sweat gland. *Pflügers Arch.*, **349**, 63.

LONG, C. (Ed.) (1961). *Biochemists' Handbook* p. 921. London: Spon.

McGEE, J. O'D., WOODS, J.C., ASHALL, F., BRAMWELL, M.E. & HARRIS, H. (1982). A new marker for human cancer cells. 2. Immunohistochemical detection of the Ca antigen in human tissues with the Ca1 antibody. *Lancet*, **ii**, 7.

MASTROIANNI, L., WINTERNITZ, W.W. & LOWI, N.P. (1958). The *in vitro* metabolism of the human endosalpinx. *Fertil. Steril.*, **9**, 500.

MEYER, K.A., KAMMERLING, E.M., AMTMAN, L., KOLLER, M. & HOFFMAN, S.J. (1948). pH Studies of malignant tissues in human beings. *Cancer Res.*, **8**, 513.

PETERSON, H-I. (1979). *Tumour Blood Circulation: Angiogenesis, Vascular Morphology and Blood Flow of Experimental and Human Tumours.* (Ed. Peterson.) Florida, USA: CRC Press Inc.

RESTALL, B.J. & WALES, R.G. (1966). The fallopian tube of the sheep. III. The chemical composition of the fluid from the fallopian tube. *Aust. J. Biol. Sci.*, **19**, 687.

SPIRO, R.G. & BHOYROO, V.D. (1974). Structure of the 0-glycosidically linked carbohydrate units of fetuin. *J. Biol. Chem.*, **249**, 5704.

UMEMOTO, J., BHAVANANDAN, V.P. & DAVIDSON, E.A. (1977). Purification and properties of an endo-α-N-acetyl-D-galactosaminidase from *Diplococcus pneumoniae*. *J. Biol. Chem.*, **252**, 8609.

VAUPEL, P.W., FRINAK, S. & BICHER, H. (1981). Heterogeneous oxygen partial pressure and pH distribution in C3H mouse mammary adenocarcinoma. *Cancer Res.*, **41**, 2008.

WILLIAMS, A.F. (1977). Differentiation antigens of the lymphocyte cell surface. *Contemp. Topics Molec. Immunol.*, **6**, 83.

WOODS, J.C., SPRIGGS, A.J. HARRIS, H. & McGEE, J. O'D. (1982). A new marker for human cancer cells. 3. Immunocytochemical detection of malignant cells in serous fluids with the Ca1 antibody. *Lancet*, **ii**, 512.